# An Insight into FDA Approved Antibody-Drug Conjugates for Cancer Therapy

**DOI:** 10.3390/molecules26195847

**Published:** 2021-09-27

**Authors:** Juliana T. W. Tong, Paul W. R. Harris, Margaret A. Brimble, Iman Kavianinia

**Affiliations:** 1 School of Chemical Sciences, The University of Auckland, Auckland 1010, New Zealand; jton210@aucklanduni.ac.nz (J.T.W.T.); paul.harris@auckland.ac.nz (P.W.R.H.); 2 Maurice Wilkins Centre for Molecular Biodiversity, The University of Auckland, Auckland 1010, New Zealand; 3 School of Biological Sciences, The University of Auckland, Auckland 1010, New Zealand

**Keywords:** antibody-drug conjugates, ADCs, targeted therapy, cancer, FDA approved

## Abstract

The large number of emerging antibody-drug conjugates (ADCs) for cancer therapy has resulted in a significant market ‘boom’, garnering worldwide attention. Despite ADCs presenting huge challenges to researchers, particularly regarding the identification of a suitable combination of antibody, linker, and payload, as of September 2021, 11 ADCs have been granted FDA approval, with eight of these approved since 2017 alone. Optimism for this therapeutic approach is clear, despite the COVID-19 pandemic, 2020 was a landmark year for deals and partnerships in the ADC arena, suggesting that there remains significant interest from Big Pharma. Herein we review the enthusiasm for ADCs by focusing on the features of those approved by the FDA, and offer some thoughts as to where the field is headed.

## 1. Introduction

Paul Ehrlich’s vision of a rationally targeted strategy to eliminate disease, whether it be microbes or malignant cells, has driven research over the past century, particularly creating a targeted cancer therapy revolution [1]. In 1913, it was theorized that a so-called ‘magic bullet’ drug could cause selective destruction by employing a toxin and a targeting agent. Over 80 years following Ehrlich’s fundamental realization, and supported by the successful development of chemotherapy in the 1940s [2] and monoclonal antibodies (mAbs) in the 1970s [3], in 1983 the first successful antibody-drug conjugate (ADC) human clinical trial began using an anti-carcinoembryonic antibody tethered to vindesine [4]. The safety of administration and the ability of the conjugate to localize after radiolabeling was investigated in eight patients with advanced metastatic carcinoma. While the feasibility of this approach was demonstrated, several hurdles were identified, the most significant being aggregation [4]. 

**Figure 1:**
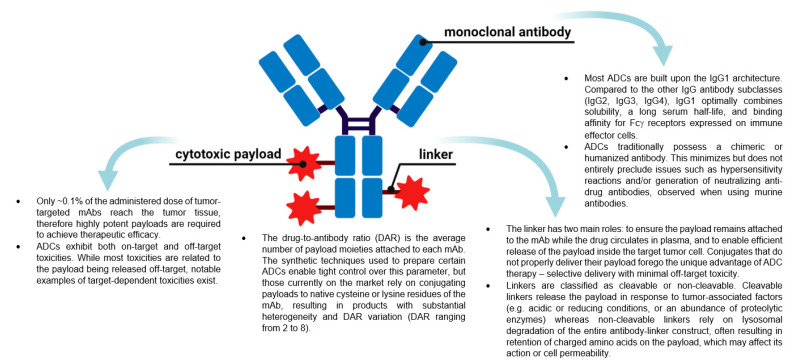
The general structure of an antibody-drug conjugate (ADC) and key points about the different components. (Created with BioRender.com, accessed 27 September 2021).

ADCs are now amongst the fastest growing drug classes in oncology, as they combine the best features of mAbs and small molecule drugs, creating a single moiety that is highly specific and cytotoxic. These therapeutic entities are considered the “homing missiles” of cancer therapy, and are composed of three key elements: a monoclonal antibody that selectively binds to an antigen on the tumor cell surface, a cytotoxic drug payload, and a cleavable or non-cleavable linker, see Figure 1 [5,6,7]. Each of these components can vary widely between ADCs, leading to immense diversity in the overall structure, and subsequently, the ADC’s pharmacological and clinical properties. ADCs are designed to deliver the toxic payload selectively to cells expressing the target antigen. Therefore, target antigens that are preferentially expressed in tumors versus non-malignant cells can be exploited to harness a greater therapeutic window and reduce the chance of off-target effects associated with systemic administration of traditional chemotherapeutics. The advent of ADCs has thus sparked a revival of chemotherapeutic payloads, which cannot be administered systemically due to their extreme potency and ensuing toxicity profiles.

**Figure 2:**
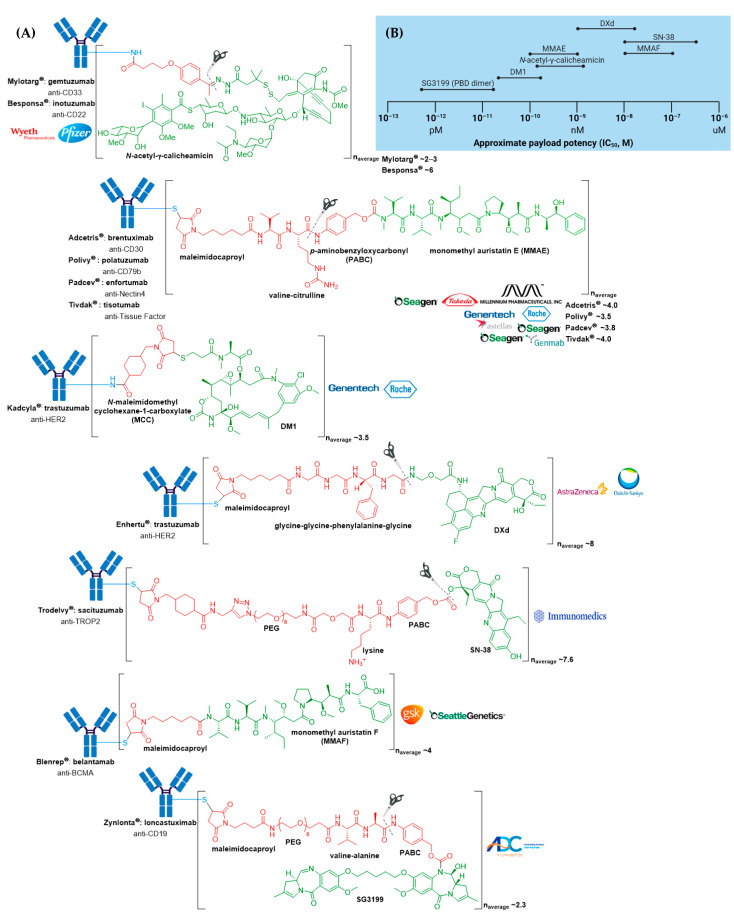
(**A**) Structures of FDA approved antibody-drug conjugates (ADCs). The antibody is shown in blue, and chemical structures for linker and payload are in red and green, respectively. Scissors indicate the cleavage site (if applicable). Pharmaceutical makers and drug-to-antibody ratio for each ADC is indicated. (**B**) Comparison of approximate payload potency ranges (Created with BioRender.com, accessed September 2021).

Many ADCs have demonstrated impressive activity against treatment-refractory cancers, resulting in their approval for both hematologic malignancies and solid tumor indications. At the time of writing, 11 different ADCs have been approved by the US Food and Drug Administration (FDA) for clinical use, see Figure 2A and Table 1. Of these, seven have also obtained approval by the European Medicines Agency (EMA) (Appendix A). The recent surge in ADC approvals, of which Polivy^®^ (polatuzumab vedotin-piiq), Padcev^®^ (enfortumab vedotin-ejfv), Enhertu^®^ (fam-trastuzumab deruxtecan-nxki), Trodelvy^®^ (sacituzumab govitecan-hziy), Blenrep^®^ (belantamab mafodotin-blmf), Zynlonta^®^ (loncastuximab tesirine-lpyl), and Tivdak^®^ (tisotumab vedotin-tftv) have all gained FDA approval since 2019, belies the turbulent past these biologics have experienced, both in academic and regulatory settings. 

While several publications have listed Lumoxiti^®^ (moxetumomab pasudotox-tdfk) as an FDA approved ADC [8,9], we have excluded it from our discussions as we consider it an immunotoxin [10,11,12,13]. Besides Lumoxiti^®^ [14,15], the immunotoxins Ontak^®^ (denileukin diffittox) [16] and Elzonris^®^ (tagraxofusp-erzs) [17], have also been granted FDA approval.

**Figure 3:**
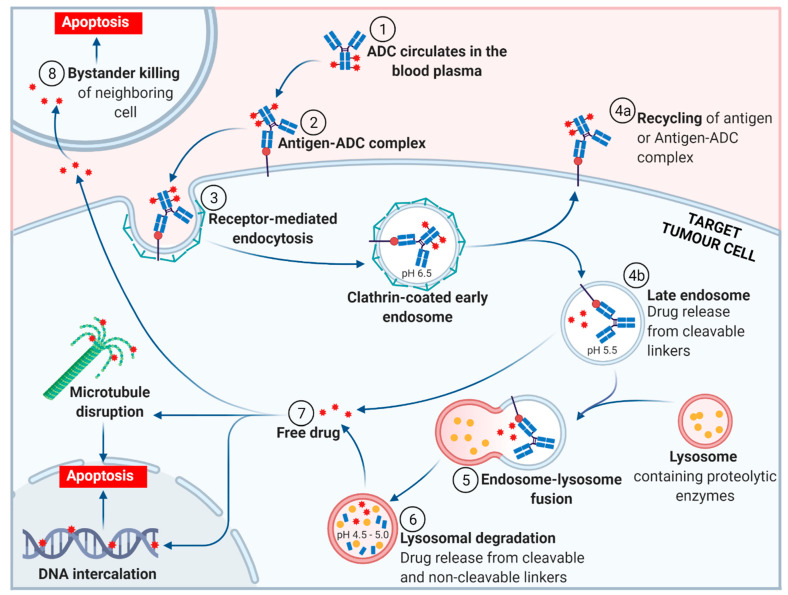
The general mechanism of action of an antibody-drug conjugate (ADC). (Adapted from “Antibody-Drug Conjugate Release”, by BioRender.com (accessed 27 September 2021). Retrieved from https://app.biorender-templates, accessed 27 September 2021).

In this review, we aim to provide a brief and up to date overview of each of the FDA approved ADCs. We begin with the general mechanism of action (MoA) of an ADC, see Figure 3, followed by a chronological discussion of the FDA approved ADCs (based on year of first approval). References to pivotal clinical studies leading to approval are included. We conclude with our thoughts on where the ADC field is headed, particularly focusing on expected market growth and the use of artificial intelligence (AI) to drive the development of ADC technologies. Literature documenting ADCs is extensive, with over 60,000 research articles pertaining to ADCs published between 2011 and 2018 [18]. We recommend several excellent review articles in the field of ADCs for more detail and to promote the understanding and an appreciation of these next-generation therapeutics [19,20,21,22,23,24].

## 2. ADC Mechanism of Action

The general mechanism of action for an ADC is depicted in Figure 3. Following the introduction of the ADC into the plasma circulation (step 1), recognition of a specific antigen on the tumor cell surface leads to strong binding and formation of an antigen–ADC complex (step 2). The entire complex is internalized, predominantly through receptor-mediated endocytosis with formation of a clathrin-coated early endosome (step 3) [25]. Inside the early endosome, some ADCs bind neonatal Fc receptors (FcRns) and undergo transcytosis to the extracellular space (step 4a) [25,26]. Following endosomal maturation to a late endosome, characterized by an environment with low luminal pH [27], those ADCs retained in the endosome undergo drug release from cleavable linkers (step 4b). The late endosome fuses with a lysosome (step 5), inside which the ADC and/or its components are exposed to proteolytic enzymes (e.g., cathepsin B) and an increasingly acidic environment, promoting further payload release (step 6). The free drug then exerts its cellular destruction via a pathway specific to the mode of action of the payload. Most ADC payloads cause apoptosis by DNA damage or microtubule disruption (step 7). In addition, some payloads (those sufficiently hydrophobic to cross cell membranes and initially tethered to an antibody via a cleavable linker) exert a bystander effect. Free drug is exported from the target tumor cell, across the cell membrane to kill neighboring tumor cells, including those that may not express the relevant antigen on its cell surface or are less accessible directly from the circulatory system (step 8).

## 3. FDA Approved ADCs

### 3.1. Mylotarg^®^

**Figure 4:**
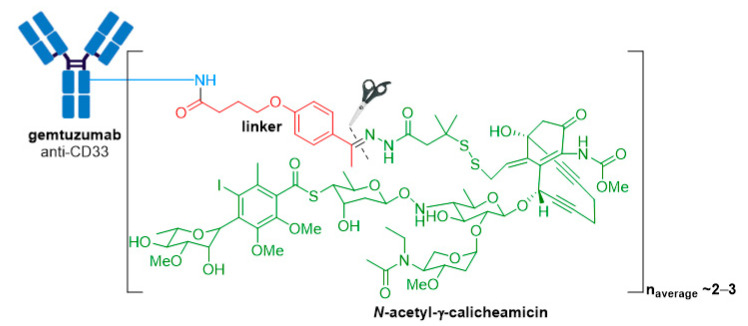
Structure for Mylotarg^®^ (gemtuzumab ozogamicin). The antibody is shown in blue, and chemical structures for linker and payload are in red and green, respectively. The cleavage site is indicated by scissors.

Mylotarg^®^ (gemtuzumab ozogamicin) from Wyeth/Pfizer was the first ADC to reach the market. It is composed of a recombinant humanized anti-CD33 mAb (IgG4κ antibody hP67.6) covalently attached to a calicheamicin derived payload (*N*-acetyl-γ-calicheamicin 1,2-dimethyl hydrazine dichloride) via a pH-sensitive hydrazone linker, see Figure 4 [28,29].

Highlighting the rocky start for ADC therapeutics, Mylotarg^®^ was granted accelerated approval for relapsed CD33+ acute myeloid leukemia (AML) in 2000, but was voluntarily withdrawn from the market in 2010 after post-approval studies failed to verify survival benefit and demonstrated a higher rate of fatal toxicity than chemotherapy alone [30,31]. Despite this, Mylotarg^®^ was reapproved by the FDA in 2017 under an alternative dosing regimen (previously administered as one dose of 9 mg/m^2^) of three doses of 3 mg/m^2^, and a different patient population was introduced [32]. These changes reduced the maximum plasma concentration, thus improving the safety profile and response rate when administered as a single-agent [33,34] or combination regimen [35,36].

**Figure 5:**
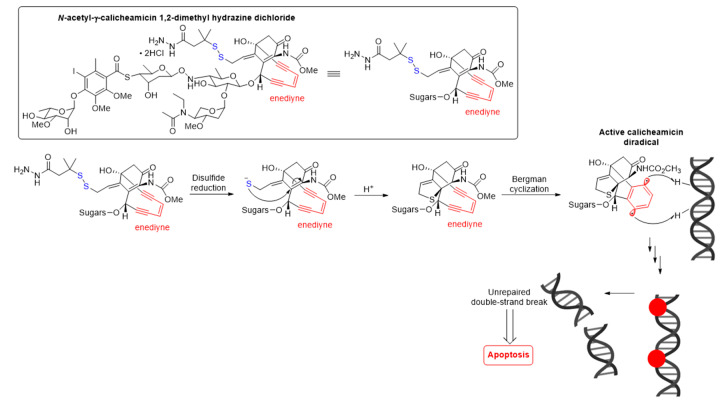
Mechanism for double-strand (ds) DNA cleavage by *N*-acetyl-γ-calicheamicin. The enediyne warhead is shown in red.

The antitumor activity of Mylotarg^®^ results from the semi-synthetic payload, a calicheamicin derivative (*N*-acetyl-γ-calicheamicin 1,2-dimethyl hydrazine dichloride) produced by microbial fermentation followed by synthetic modification. The payload consists of four glycosidic units, a fully substituted iodobenzoate moiety, and an aglycon. The highly reactive hex-3-ene-1,5-diyne subunit can be readily triggered to aromatize via a Bergman cyclization reaction, generating a benzene-1,4-diradical [37]. This aromatization process affords a resulting diradical that can abstract two hydrogen atoms from the DNA backbone, leading to unrepairable double-strand (ds) DNA breaks followed by cell-cycle arrest and apoptotic cell death, see Figure 5 [37].

A crucial feature for successful construction of an ADC is the conjugation chemistry of the linker-payload with the mAb. In Mylotarg^®^, the bifunctional 4-(4-acetylphenoxy)butanoic acid moiety provides attachment to surface-exposed lysine residues of the mAb through an amide bond, and the linker forms an acyl hydrazone linkage with the payload. Mylotarg^®^ is considered a first-generation ADC because it utilizes *N*-hydroxysuccinimide chemistry to conjugate calicheamicin to surface-exposed lysine residues on the antibody, yielding a heterogenous mixture with different drug-to-antibody ratios (DARs) [38]. The number of conjugated calicheamicin derivatives per mAb ranges from zero to six, with an average drug loading of two to three molecules of calicheamicin per antibody.

The acid-cleavable hydrazone linker is designed to be stable in the neutral pH conditions encountered during circulation, however, hydrolysis is readily achieved under the acidic environment of lysosomes (pH ~4.5–5.0) inside CD33+ target cells. The dimethyl disulfide moiety preserves the natural disulfide trigger mechanism of calicheamicin, while the added steric hindrance resulting from the methyl substituents protects the disulfide from reduction during circulation [38,39].

As for all humanized antibodies, complementarity determining region (CDR) grafting was used for humanization of the anti-CD33 murine antibody, hP67.6, employed in Mylotarg^®^ [40,41]. The resulting antibody is a genetically engineered IgG4κ antibody containing sequences derived from the murine antibody, but with an increased similarity to antibody variants produced naturally in humans. While the IgG4 antibody isotype has the longest circulating half-life of all isotypes, it is least likely to participate in immune-mediated mechanisms, such as complement fixation and antibody-dependent cellular cytotoxicity (ADCC) [42]. Although antibody effector functions, such as ADCC, complement-dependent cytotoxicity, and antibody-dependent cellular phagocytosis (ADCP), have the potential to augment antitumor activities, engaging Fcγ receptors can also lead to increased off-target and dose-limiting toxicity [43,44,45]. Several next-generation ADCs have thus exploited antibody engineering to enhance or impair immune effector functions.

Demonstrating that failure is perhaps merely a step towards success, the pitfalls and limitations of this first-generation ADC provided several key lessons for future improvements in ADC research.

### 3.2. Adcetris^®^

**Figure 6:**
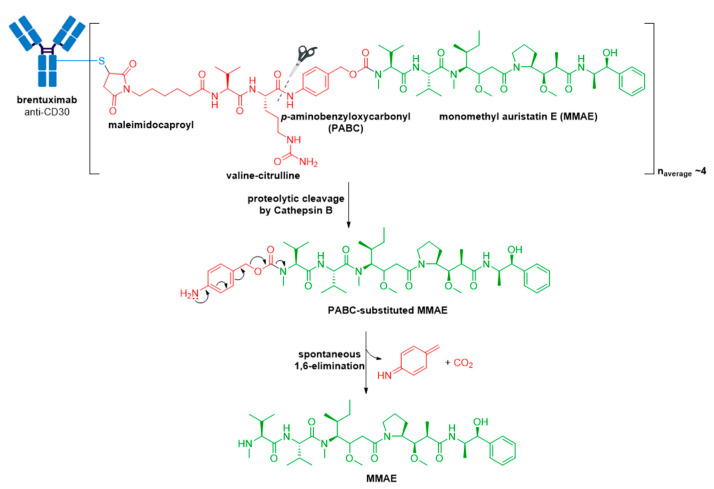
Structure of Adcetris^®^ (brentuximab vedotin). The antibody is shown in blue, and chemical structures for linker and payload are in red and green, respectively. Spontaneous 1,6-elimination mechanism for the PABC-substituted MMAE, leading to release of MMAE, *p*-iminoquinone methide, and carbon dioxide.

Adcetris^®^ (brentuximab vedotin) from Seagen (formerly Seattle Genetics), containing a CD30-specific mAb conjugated to monomethyl auristatin E (MMAE), received FDA approval in 2011, making it the second ADC to enter the oncology market, see Figure 6 [46,47,48,49]. It is approved for Hodgkin lymphoma (HL) [50,51] and systemic anaplastic large cell lymphoma (sALCL) [52] in the USA, Europe, and Japan [47,53].

The anticancer activity of Adcetris^®^ results from the binding of MMAE to tubulin. This disrupts the microtubule network within the cell, subsequently inducing cell cycle arrest and apoptotic cell death [54]. In addition, likely owing to the IgG1 antibody isotype, in vitro data provide evidence for ADCP antitumor activity [55]. From first-generation ADCs, it was learnt that ~0.1% of the injected ADC dose reaches the target tumor site, thus necessitating an increase in potency of the cytotoxic agent and/or DAR for improved therapeutic activity [56,57]. Adcetris^®^ addressed these two requirements by employing the more cytotoxic payload MMAE, a tubulin-targeting agent, belonging to the auristatin family of drug payloads (cytotoxicity in the low nanomolar to sub-nanomolar range against a variety of cancer types). See Figure 2B for a comparison of approximate cytotoxicity ranges (based on concentrations giving 50% maximum inhibition, IC_50_) for payloads employed in FDA approved ADCs. Furthermore, as compared to Mylotarg^®^ with a DAR of two to three, Adcetris^®^ has approximately four molecules of MMAE attached to each antibody molecule. 

The pitfall of premature drug release resulting from the acid-cleavable hydrazone linker in Mylotarg^®^ [20] was addressed in the second-generation ADC, Adcetris^®^, by using the protease-cleavable “mc-vc-PABC-MMAE” linker-drug combination [48,49,58,59,60]. This linker construct utilizes a thiol-reactive maleimidocaproyl (mc) spacer, a valine-citrulline (vc) dipeptide, and a self-immolative *para*-aminobenzyloxycarbonyl (PABC) spacer [60]. The mc spacer is incorporated for conjugation to cysteine residues of the mAb, and a PABC spacer allows linker attachment to the secondary amine of MMAE. Due to the steric bulk of the payload, the PABC spacer also facilitates enzyme access allowing the vc group to be recognized by cathepsin B [20,60,61]. Cathepsin B is a cysteine protease which presents almost exclusively in the lysosomal compartment in healthy mammals, and is overexpressed in multiple cancer types [62,63]. It is responsible for cleaving the citrulline-PABC amide bond. Following proteolytic cleavage, the resultant PABC-substituted MMAE forms an unstable intermediate which spontaneously undergoes a 1,6-elimination with loss of *p*-iminoquinone methide and carbon dioxide to release the free drug, see Figure 6.

Compared to Mylotarg^®^, which uses an IgG4 antibody, the IgG subclass employed in Adcetris^®^ is IgG1. This is the most common subclass for ADCs, as while having similarly long serum half-lives to IgG4, they possess greater complement-fixation and FcγR-binding efficiencies [42].

**Figure 7:**
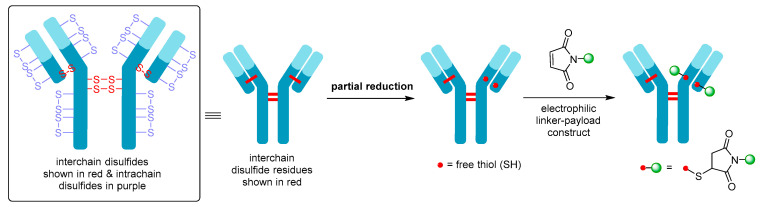
Schematic showing partial reduction of IgG1 antibody interchain disulfide bonds to generate two nucleophilic free thiol groups that can be reacted with an electrophilic linker-payload construct, such as maleimide (DAR = 2). Maleimide conjugation to cysteine is shown in this example.

Although Mylotarg^®^ utilizes lysine residues on the mAb for linker-payload conjugation, Adcetris^®^ employs cysteine-based conjugation. Due to the limited number of cysteine conjugation sites available (four interchain and twelve intrachain disulfides, see Figure 7, as opposed to 80–100 lysine amines for IgG1) and the distinct reactivity of thiols, this approach enables improved homogeneity of the ADC species and a more controlled drug loading [21]. Cysteine conjugation relies on partial or full reduction of the four interchain disulfides to produce an average number (e.g., two, four, six, or eight) of free nucleophilic thiols, while keeping the intrachain disulfide bonds intact. Interchain disulfides are generally not critical for structural stability and have higher solvent accessibility, making them an ideal target. They are typically reduced using reagents such as tris(2-carboxyethyl)phosphine (TCEP), dithiothreitol (DTT), or 2-mercaptoethylamine (2-MEA) prior to conjugation [21]. Once the free thiols are generated, they can be reacted with a linker-payload complex possessing a suitable electrophilic group, see Figure 7. Maleimide chemistry has been the mainstay for linkage to cysteines, with all auristatin-containing ADCs utilizing the maleimidocaproyl (mc) linkage to the antibody [61].

Although an improvement over lysine conjugation, this method still produces a heterogenous mixture of ADC species, which can negatively impact on parameters including pharmacokinetics, tolerability, and efficacy [18]. Therefore, site-specific conjugation methodologies have been developed, of which THIOMAB^TM^ technology is the most well-known [64,65]. Genentech’s THIOMAB^TM^ antibody platform uses site-directed mutagenesis to incorporate cysteine residues into antibodies at positions on light and heavy chains that provide reactive thiol groups without perturbing immunoglobulin folding and assembly, or altering antigen binding [64,65]. Although homogenous ADCs have repeatedly demonstrated superior overall pharmacological profiles compared to their heterogenous counterparts, engineered antibodies for site-specific conjugation have not yet been employed in any of the FDA approved ADCs. We recommend the review by Walsh and co-workers for an in-depth understanding of chemical and enzymatic methods for site-specific antibody modification, resulting in the generation of homogenous ADCs [21].

### 3.3. Kadcyla^®^

In 2013, Kadcyla^®^ (ado-trastuzumab emtansine), developed and marketed by Genentech/Roche, revolutionized the field of ADCs by becoming the first ADC approved for the treatment of solid tumors. It is indicated as an adjuvant (after surgery) treatment for HER2+ early breast cancer in patients who previously received trastuzumab (Herceptin^®^) and a taxane, separately or in combination [66,67,68,69].

**Figure 8:**
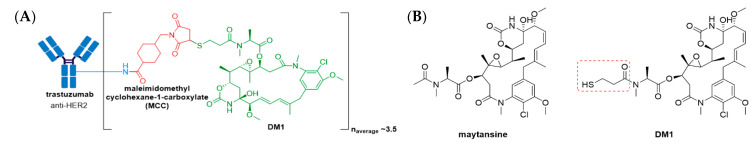
(**A**) Structure of Kadcyla^®^ (ado-trastuzumab emtansine). The antibody is shown in blue, and chemical structures for linker and payload are in red and green, respectively. (**B**) The chemical structure for maytansine and DM1. The thiopropanoyl group of DM1, which allows for conjugation to a maleimidomethyl cyclohexane-1-carboxylate (MCC) group is shown in the red box.

This approval marked a milestone achievement in ADC development because effective treatment of solid tumors using such therapy previously posed a formidable challenge. Firstly, prior to Kadcyla^®^, the treatment of solid tumors with ADCs fell short due to numerous biological barriers in the tumor microenvironment (e.g., poor vascularization, diffusion through dense stroma, overcoming tumor interstitial fluid pressure) which limited drug penetration. Secondly, unlike hematologic malignancies, the concept of lineage-specific antigen expression is not applicable to solid tumors, for which the antigens expressed are mainly “tumor associated” rather than “tumor specific” [70]. This implies both a share of on-target/off-tumor toxicity and thus reduced intra-tumoral drug delivery. Kadcyla^®^ comprises the humanized anti-HER2 IgG1 antibody, trastuzumab, linked to the antimitotic agent, DM1, see Figure 8A [69,71]. DM1 is a potent maytansine derivative, belonging to the maytansinoid family of natural products. While maytansine is difficult to conjugate due to the absence of reactive functional groups, DM1 contains a thiopropanoyl group instead of the native *N*-acetyl group, see Figure 8B, allowing for lysine-antibody conjugation via a non-reducible thioether linker, maleimidomethyl cyclohexane-1-carboxylate (MCC).

Compared to the two previously mentioned FDA approved ADCs, Kadcyla^®^ consists of a non-cleavable thioether linker. Non-cleavable linkers tend to be more stable than their cleavable counterparts, but they rely on lysosomal degradation of the entire antibody-linker construct for payload release. This often results in retention of charged amino acids on the payload, which may affect its action or cell permeability. In human plasma, Kadcyla^®^ catabolites, MCC-DM1, lysine-bound emtansine (Lys-MCC-DM1), and DM1 have been detected at low levels. Cytotoxic effects of Kadcyla^®^ result from DM1-containing catabolites (primarily Lys-MCC-DM1) binding to tubulin, which disrupts microtubule networks, inducing cell cycle arrest and apoptotic cell death at sub-nanomolar concentrations [72]. In addition, in vitro studies have shown that Kadcyla^®^ mediates ADCC [69].

Undoubtedly, the approval of Kadcyla^®^ in 2013 was a big win for Swiss drug maker, Roche. In 2019, annual sales surpassed US$1 billion, deeming Kadcyla^®^ the first ADC to achieve blockbuster status.

### 3.4. Besponsa^®^

**Figure 9:**
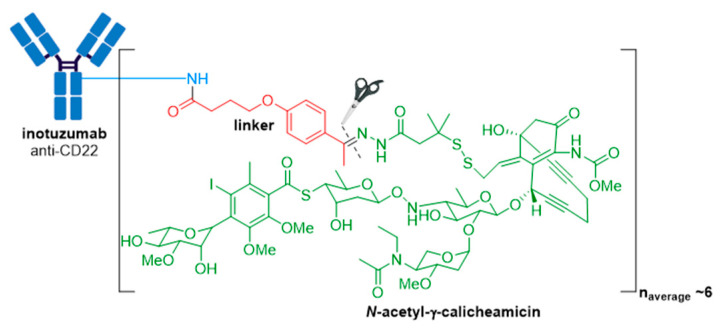
Structure of Besponsa^®^ (inotuzumab ozogamicin). The antibody is shown in blue, and chemical structures for linker and payload are in red and green, respectively.

Besponsa^®^ (inotuzmab ozogamicin (Pfizer/Wyeth)) obtained FDA approval in 2017 and is directed against CD22+ B-cell acute lymphoblastic leukemia (B-ALL) [73,74,75]. It is based on an ADC platform similar to Mylotarg^®^ (see Section 3.1) (Figure 9) [74,75]. The first difference lies in the mAb and thus the antigen target and cancer indication. The recombinant humanized monoclonal IgG4 antibody (G544) employed in Besponsa^®^ is selective for CD22 expressed on B cells in all patients with mature B-ALL, and >90% of patients with precursor B-ALL. In addition, preclinical studies demonstrated Besponsa^®^ could tolerate a higher DAR of ~6 (*cf.* Mylotarg^®^ 2–3) without significant aggregation [75].

### 3.5. Polivy^®^ and Padcev^®^

**Figure 10:**
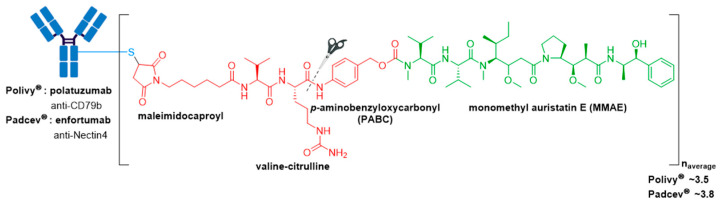
Structure of Polivy^®^ (polatuzumab vedotin-piiq) and Padcev^®^ (enfortumab vedotin-ejfv). The antibody is shown in blue, and chemical structures for linker and payload are in red and green, respectively.

Highlighting the importance of antigen selection and thus the mAb for targeted drug delivery, both Polivy^®^ (polatuzumab vedotin-piiq) and Padcev^®^ (enfortumab vedotin-ejfv) possess the same mc-vc-PABC-MMAE linker-drug construct as Adcetris^®^ (see Section 3.2) (Figure 10) [76,77]. Both ADCs were approved by the FDA in 2019. 

Polivy^®^ is an anti-CD79b ADC developed by Genentech/Roche using a proprietary technology developed by Seagen [78]. It is indicated in combination with bendamustine and rituximab for treatment of adults with relapsed or refractory diffuse large B-cell lymphoma (DLBCL), an aggressive type of non-Hodgkin lymphoma, who have received at least two prior therapies [76,79]. This indication was granted accelerated approval based on a complete response rate. Polivy^®^ has an approximate DAR of 3.5 molecules of MMAE attached to each antibody.

Padcev^®^, produced and marketed by Astellas Pharma Inc. and Seagen is a Nectin4-directed ADC [80]. It was first granted accelerated approval in 2019 for treatment of adults with locally advanced or metastatic urothelial cancer who have previously received a programmed death receptor-1 (PD-1) or programmed death-ligand 1 (PD-L1) inhibitor, and a platinum-containing therapy [81]. In 2021, this indication was granted regular approval and Padcev^®^ was granted accelerated approval for patients which are ineligible for cisplatin-containing chemotherapy and have previously received one or more prior lines of therapy [82,83]. Padcev^®^ is comprised of a fully humanized anti-Nectin4 IgG1κ mAb (AGS-22C3) produced by mammalian (Chinese hamster ovary) cells, and has an approximate DAR of 3.8.

### 3.6. Enhertu^®^

Enhertu^®^ (fam-trastuzumab deruxtecan-nxki), developed by Daichi Sankyo/AstraZeneca, was granted accelerated FDA approval in December 2019 for treatment of adult patients with unresectable or metastatic HER2+ breast cancer who have received two or more prior anti-HER2 based regimens [84,85]. Furthermore, in 2020, the FDA granted this ADC breakthrough therapy designation for treatment of patients with metastatic, HER2-mutated non-small cell lung cancer (NSCLC) after a platinum-based therapy, and priority review for treatment of HER2+ metastatic gastric or gastroesophageal junction adenocarcinoma.

Showcasing the continued promise of Enhertu^®^, in 2021 the ADC was approved in the US for a second oncology indication treatment of adult patients with locally advanced or metastatic HER2+ gastric or gastroesophageal junction adenocarcinoma, who have received a prior trastuzumab-based regimen [85,86].

**Figure 11:**
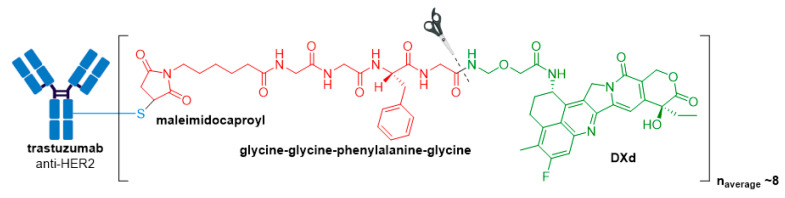
Structure of Enhertu^®^ (fam-trastuzumab deruxtecan-nxki). The antibody is shown in blue, and chemical structures for linker and payload are in red and green, respectively.

The ADC is comprised of an anti-HER2 antibody, a protease cleavable tetrapeptide-based linker, and DXd as the drug payload, see Figure 11 [85,87]. DXd is a novel exatecan derivative designed using Daiichi Sankyo’s proprietary ADC technology. It belongs to the camptothecin class of drug payloads, which cause their cytotoxic effects by inhibiting topoisomerase I (TOP1) enzyme. TOP1 is essential in higher eukaryotes as it is responsible for relaxing DNA supercoiling generated by transcription, replication, and chromatin remodeling [88]. Therefore, inhibition of this enzyme leads to DNA damage and apoptotic cell death, resulting in destruction of HER2+ tumor cells.

Besides the potent warhead, several biochemical improvements differentiate Enhertu^®^ from the previously approved anti-HER2 ADC, Kadcyla^®^. Firstly, the DAR of Enhertu^®^ is more homogenous and approximately twice that of Kadcyla^®^ (8 vs. 3–4), thereby leading to an increased drug concentration inside target tumor cells [87]. Secondly, the drug and antibody are connected via a novel cathepsin-cleavable peptide linker. The linker is connected to a cysteine residue of the antibody via a maleimidocaproyl group, and the tetrapeptide portion consisting of the amino acid sequence, glycine-glycine-phenylalanine-glycine, attaches to the proprietary payload by an amide bond. The hydrophobic nature of this payload improves cell membrane permeability, thus maximizing bystander killing effects of the ADC, and deeming it effective against HER2-negative cells.

Following the initial success of Enhertu^®^, Daiichi Sankyo and AstraZeneca signed a $6 billion deal to develop and commercialize other ADCs based on the same technology [89,90]. According to the terms of the agreement, Daiichi Sankyo will receive $1 billion in staged payments from AstraZeneca and the Japanese company will also be eligible for up to $1 billion for regulatory milestones and $4 billion for sales-related milestones [89,90]. This agreement represents the second collaboration between Daiichi Sankyo and AstraZeneca, reflects AstraZeneca’s continued strategy to invest in ADCs as a class, the innovative nature of the technology, and the successful existing collaboration with Daiichi Sankyo.

### 3.7. Trodelvy^®^

Further highlighting the industry’s appetite for ADC technology, in October 2020, Gilead Sciences paid $21 billion to acquire Immunomedics, and its recently approved ADC, Trodelvy^®^ (sacituzumab govitecan-hziy) [91]. In April 2020, Trodelvy^®^ received accelerated FDA approval for treatment of patients with locally advanced or metastatic triple-negative breast cancer (mTNBC) who have received at least two prior therapies for metastatic disease [92,93]. Demonstrating its commercial success, Trodelvy^®^ recorded $US20 million in sales in its first two months on the market, and generated sales for the fourth quarter and full year 2020 (including the period prior to the completion of Gilead’s acquisition of Immunomedics) of $64 million and $137 million, respectively [94]. In April 2021, the FDA granted regular approval for this indication, and in the same year, Trodelvy^®^ was granted accelerated approval for a second indication treatment of locally advanced or metastatic urothelial cancer after a platinum-containing chemotherapy and either a PD-1 or PD-L1 inhibitor [93,95].

**Figure 12:**
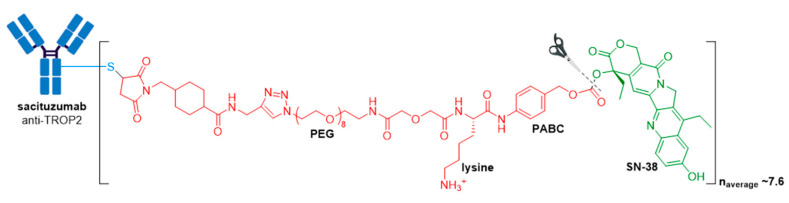
Structure of Trodelvy^®^ (sacituzumab govitecan-hziy). The antibody is shown in blue, and chemical structures for linker and payload are in red and green, respectively. PEG, polyethyleneglycol.

Trodelvy^®^ consists of a fully humanized hRS7 IgG1κ antibody targeted against TROP2 (trophoblast antigen 2) conjugated to SN-38, the active metabolite of irinotecan [88] via an acid-sensitive hydrolysable linker called CL2A, see Figure 12 [93,96,97].

This ADC is another example of an ADC with a high DAR, consisting of approximately 7.6 SN-38 molecules per antibody, which does not affect its binding and pharmacokinetics. This is allowed by the moderately toxic topoisomerase 1 inhibitor (SN-38 IC_50_ in the low micromolar range against several cancer types), and a non-stable linker prone to drug leakage and subsequent bystander effects. In a study by Goldenberg and co-workers, it was found that this ADC targets up to 136-fold more SN-38 to a human cancer xenograft than irinotecan [98]. Furthermore, since Trodelvy^®^ delivers SN-38 in its most active, non-glucuronidated form, this may explain the improved toxicity profile, as shown by a lower frequency of severe diarrhea than with irinotecan. Pegylation and the incorporation of a lysine residue in the linker system is thought to reduce ADC aggregation. The use of moderately toxic payloads is being further investigated as a method to increase payload concentration and overcome the challenges of stability and efficacy with higher DAR ADCs.

### 3.8. Blenrep^®^

**Figure 13:**
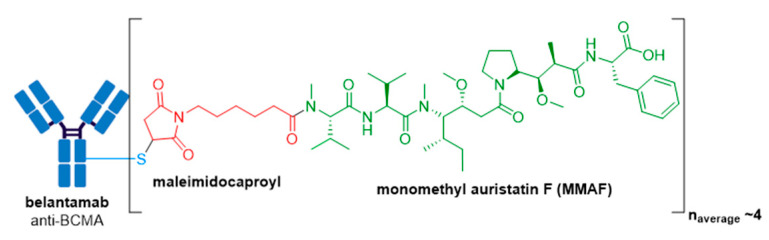
Structure of Blenrep^®^ (belantamab mafodotin-blmf). The antibody is shown in blue, and chemical structures for linker and payload are in red and green, respectively.

GlaxoSmithKline’s ADC, Blenrep^®^ (belantamab mafodotin-blmf), is the first approved anti-BCMA (B-cell maturation antigen) therapy [99]. It was granted accelerated FDA approval in August 2020 for treatment of adult patients with relapsed or refractory multiple myeloma who have received at least four prior therapies, including an anti-CD38 mAb, a proteasome inhibitor, and an immunomodulatory agent [100,101,102]. Blenrep^®^ consists of an afucosylated humanized IgG1 mAb conjugated to the tubulin inhibitor, monomethyl auristatin F (MMAF) via a non-cleavable maleimidocaproyl linker, see Figure 13 [100,103]. In addition to MMAF-induced apoptosis, secondary antitumor activity results from tumor cell lysis through ADCC and ADCP effector functions [100]. Besides Kadcyla^®^, currently this is the only other FDA approved ADC to possess a non-cleavable linker. The drug-linker technology is licensed from Seagen and the Fc-engineered afucosylated mAb is produced using Potelligent^®^ Technology licensed from BioWa. The Potelligent^®^ Technology platform uses *FUT8* knockout Chinese hamster ovary cells to eliminate fucose from the Fc regions in the antibody [104]. It is well established that when an antibody has reduced amounts of fucose in its sugar chains, it has increased affinity for FcγRIIIa and thus exhibits higher ADCC activity compared to highly-fucosylated conventional antibodies [103,105,106]. To date, Blenrep^®^ is the only FDA approved ADC with an afucosylated Fc-engineered antibody.

Blenrep^®^ consists of the antimitotic auristatin payload, MMAF, which differs from MMAE bearing a phenylalanine moiety at its *C*-terminus, rather than norephedrine. Although MMAF also prevents cellular division by inhibition of tubulin polymerization, this substitution leads to attenuated antitumor activity, whereby MMAF has IC_50_ values in the range of 100–250 nM which is more than 100-fold higher than those for MMAE [107]. Although the low cell permeability of MMAF, resulting from the charged phenylalanine residue, limits its toxicity if free drug is released from the ADC prematurely, MMAF-mediated killing is restricted to the target cell and thus cannot cause bystander killing. Consequently, MMAF ADCs require high tumor expression of the target antigen for effectiveness but are more potent than vc-MMAE ADCs when targeting internalizing antigens in vitro.

### 3.9. Zynlonta^®^

Zynlonta^®^ (loncastuximab tesirine-lpyl) developed by ADC Therapeutics is a CD19-directed ADC indicated for treatment of adult patients with relapsed or refractory large B-cell lymphoma after two or more lines of systemic therapy, including diffuse large B-cell lymphoma (DLBCL), not otherwise specified DLBCL arising from low grade lymphoma, and high-grade B-cell lymphoma [108,109]. It was granted accelerated approval for medical use by the FDA in April 2021. 

**Figure 14:**
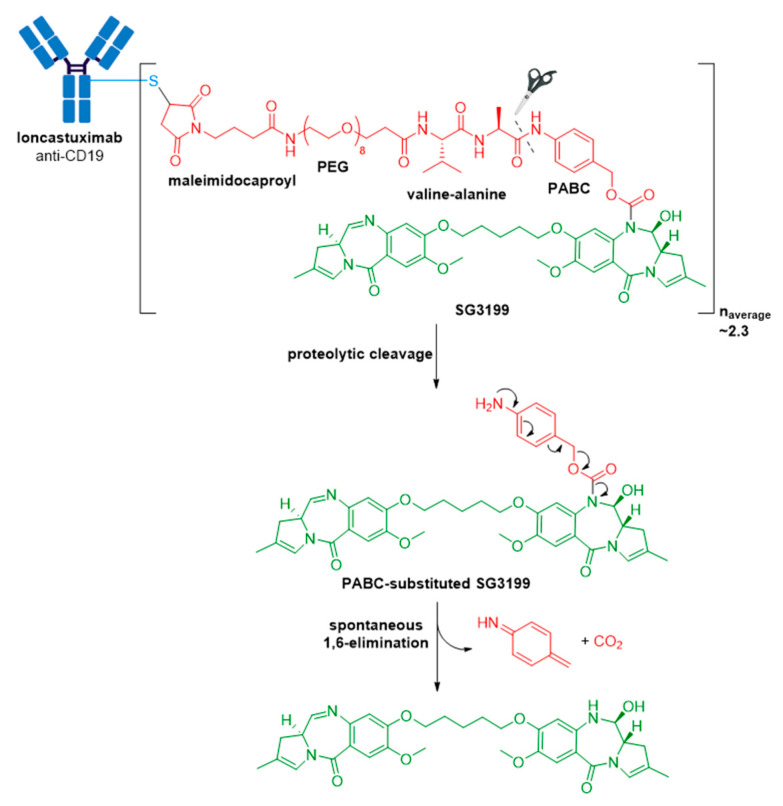
Structure of Zynlonta^®^ (loncastuximab tesirine-lpyl). The antibody is shown in blue, and chemical structures for linker and payload are in red and green, respectively. PEG, polyethyleneglycol. Spontaneous 1,6-elimination mechanism for the PABC-substituted SG3199, leading to release of SG3199, *p*-iminoquinone methide, and carbon dioxide.

**Figure 15:**
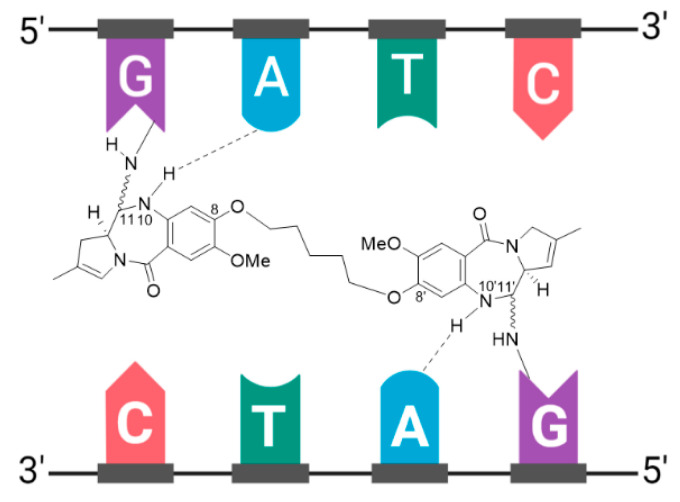
Schematic showing binding of the PBD dimer, SG3199, to the minor groove of DNA. The N2 of guanine binds the electrophilic C11 position on the PBD dimer.

Zynlonta^®^ is composed of a humanized IgG1κ mAb conjugated to SG3199, a cytotoxic pyrrolobenzodiazepine (PBD) dimer alkylating agent, through a protease-cleavable valine-alanine linker, see Figure 14 [108,110]. SG3199 exhibits cytotoxicity in the picomolar range against various cancer cell types, meaning Zynlonta^®^ possesses the most cytotoxic payload employed in a marketed ADC to date. PBD dimers are extremely potent compounds which exert their cytotoxic effects by selectively alkylating the minor groove of DNA, thereby forming adducts to inhibit nucleic acid synthesis. Following insertion in the minor groove, an aminal bond is formed through the nucleophilic attack of N2 of guanine at the electrophilic C11 position on the PBD, see Figure 15. In developing Zynlonta^®^, ADC therapeutics used the N10 position of PBD to connect the linker through a carbamate moiety. As for PABC-substituted MMAE depicted in Figure 6, PABC-substituted SG3199 undergoes a spontaneous 1,6-elimination to release the active drug, see Figure 14. Owing to the sub-picomolar potency and lipophilicity of this payload, which increases risk of toxicity in the case of premature drug release or ADC aggregation, an average of 2.3 molecules of linker-payload are attached to each mAb, and a pegylated spacer was employed. 

### 3.10. Tivdak^®^

**Figure 16:**
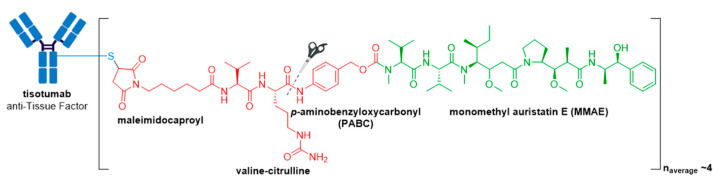
Structure of Tivdak^®^ (tisotumab vedotin-tftv). The antibody is shown in blue, and chemical structures for linker and payload are in red and green, respectively.

In late September 2021, the FDA granted accelerated approval to Tivdak^®^ (tisotumab vedotin-tftv), deeming it the most recently approved ADC on the market. Tivdak^®^, co-developed by Seagen and Genmab, is the first and only approved ADC indicated for treatment of adult patients with recurrent or metastatic cervical cancer with disease progression on or after chemotherapy [111,112]. This is the third FDA approved ADC for Seagen, further cementing their dominance as the industry leader in ADC technologies. Tivdak^®^ is a Tissue Factor (TF) directed ADC comprised of a human anti-TF IgG1κ antibody conjugated to MMAE via the same protease-cleavable mc-vc-PABC linker construct employed in Adcetris^®^, Polivy^®^, and Padcev^®^, see Figure 16 [111,113]. As for these previously discussed ADCs, Tivdak^®^ carries an average of four MMAE molecules per mAb. Furthermore, in vitro studies have demonstrated that this ADC also mediates ADCP and ADCC effector functions, thus providing multimodal antitumor activity [111].

## 4. Future Outlook and Conclusions

After decades of research and troubleshooting, appreciable technological advances and an improved mechanistic understanding of ADC activity has culminated in the FDA approval of 11 ADCs, each providing demonstrable therapeutic benefit to cancer patients. With ~297 ADCs in pre-clinical/clinical development, this suggests the world is embracing a new era of targeted cancer therapy, despite the somewhat mixed reviews that remain within academia. Market indicators suggest the global sales of currently marketed ADCs will exceed US$16.4 billion in 2026 [8]. In this analysis, Enhertu^®^ is expected to dominate the market share held by ADCs, with global sales of $6.2 billion, making it the highest selling ADC by a considerable margin (Padcev^®^ is predicted to have the second highest sales of $3.5 billion in 2026) [8]. This impressive sales forecast is high primarily because Enhertu^®^ can be used in several subsets of breast cancer (HER2+, HR+/HER2-, and triple-negative) (Appendix B) and it has an extended treatment duration [8]. Although drug development continues to be a very risky endeavor, those investing in ADC technology are finally beginning to reap the rewards from their sustained faith in this unique field of biologics. We highly expect to see more ADC approvals in the not-so-distant future, whether they be in the form of new ADCs, or label expansions of those already approved.

Arguably, the lack of variety in the MoA for payloads, linker type, and an avoidance of engineered antibodies to improve DAR homogeneity seen in the FDA approved ADCs, may suggest an “if it ain’t broke, don’t fix it” mentality. However, we believe the true potential of this pharmacological platform is only just being realized, understood, and exploited. Given the recent enthusiasm towards the role of artificial intelligence (AI) for drug discovery and development in neighboring fields, stimulated in large part by improvements in machine learning and ultimately the competitive force in the race towards the next blockbuster drug, it is presumed that drug companies will exploit these computer-based platforms for the development of next-generation ADCs [114,115,116,117,118].

As ADCs have undergone clinical development, it has become clear that the rules applying to standard chemotherapy or antibody-based therapies on their own do not necessarily apply to ADCs. ADCs are modular in nature, with interchangeable components that can be altered in a strategic fashion to improve both their efficacy and toxicity profiles. AI and other computational approaches can be used to harness the wealth of data pooled together from disparate sources (e.g., from literature, chemical or pharmacological experiments, gene studies, electronic health records), which is otherwise too vast and/or complex for humans to comprehend on their own. For many years now, this has led to the development of personalized medicines [119,120] and routine screening of virtual chemical libraries, searching for those that may match a newly discovered target [121]. Therefore, it is now envisaged that computer-aided design (e.g., *in silico* simulations and machine learning algorithms) has the potential to increase the efficiency and accuracy of completing the puzzle that is the successful three-part ADC system. These technologies may assist in identifying novel ADC constructs, perhaps with payloads and/or linker systems with unique MoAs, and could inform DAR ranges that can be tolerated (in terms of toxicity, hydrophobicity/aggregation, and size) for a particular construct. With this in mind, the importance of continuing to feed new information from the clinic to such learning systems is of vital significance. It is anticipated that AI will guide future drug and trial design, could improve the allocation of ADCs to those patients most likely to benefit from them, and may inform the selection of ideal drug targets and thus indications to treat.

To keep pace with advances in the technical design of ADCs, improvements in analytical techniques for ADC characterization and purification are also expected. Currently, UV-vis spectroscopy, chromatography, and mass spectrometry are the major techniques employed [122,123]. Hydrophobic interaction chromatography, for example, allows for separation, purification, and determination of ADC attributes including DAR, drug distribution, and content of unconjugated drugs under mild nondenaturing conditions that preserve the native ADC structure and activity [124]. It is thus envisaged that novel tools and techniques will be developed, not only to improve the efficiency and accuracy of ADC structural analysis, but also to help identify new parameters that could predict safety and efficacy outcomes. 

Furthermore, the promise of ADCs as a therapeutic approach is substantial, even going beyond the realms of cancer. Research is already underway into ADCs for treatment of non-oncological indications, including autoimmune and cardiovascular diseases, diabetes, and antimicrobial infections [125]. In fact, Seagen has initiated a Phase II clinical trial (NCT03222492) to study the safety and efficacy of Adcetris^®^ in systemic sclerosis, an autoimmune disease of the connective tissue [126]. Considering this disease poses a significant and unmet need for effective treatment options, the potential for Adcetris^®^ to alleviate symptoms is highly anticipated. With Adcetris^®^ already approved by the FDA, the risk of failure is lower because the drug already has an established safety profile in preclinical models and accumulated data from more than 10 years of clinical administration. Importantly, the repurposing of this ADC is an attractive proposition for Seagen, not only for the shorter development timeline and lower development costs, but as the current patentee they may also be eligible for extended patent protection over their product [127]. While cancer has proven the testing grounds for ADC therapies, their prospective value in other fields of medicine is becoming increasingly recognized. Given the significant increase in Big Pharma interest in the ADC space, continued growth of the ADC market is inevitable, and optimism remains for the development and marketing approval of ADCs with blockbuster potential [128,129].

## Figures and Tables

**Table 1:** FDA approved ADCs currently on the market.

ADC	Target	mAb	Linker	Payload/Payload Class	Payload Action	DAR	Disease Indication (Year of Approval)
Mylotarg^®^ (gemtuzumab ozogamicin)	CD33	IgG4	acid cleavable	ozogamicin/calicheamicin	DNA cleavage	2–3	CD33+ R/R AML (2000) ^a^
Adcetris^®^ (brentuximab vedotin)	CD30	IgG1	enzyme cleavable	MMAE/auristatin	microtubule inhibitor	4	R/R sALCL or cHL (2011)R/R pcALCL or CD30+ MF (2017); cHL, sALCL or CD30+ PTCL (2018) ^b^
Kadcyla^®^ (ado-trastuzumab emtansine)	HER2	IgG1	non-cleavable	DM1/maytansinoid	microtubule inhibitor	3.5	HER2+ metastatic breast cancer previously treated with trastuzumab & a taxane (2013); HER2+ early breast cancer after neoadjuvant taxane & trastuzumab-based treatment (2019)
Besponsa^®^ (inotuzumab ozogamicin)	CD22	IgG4	acid cleavable	ozogamicin/calicheamicin	DNA cleavage	6	R/R B-ALL (2017)
Polivy^®^ (polatuzumab vedotin-piiq)	CD79b	IgG1	enzyme cleavable	MMAE/auristatin	microtubule inhibitor	3.5	R/R DLBCL (2019) ^c,d^
Padcev^®^ (enfortumab vedotin-ejfv)	Nectin4	IgG1	enzyme cleavable	MMAE/auristatin	microtubule inhibitor	3.8	Locally advanced or metastatic urothelial cancer after a PD-1 or PD-L1 inhibitor and a Pt-containing chemotherapy (2019) or are ineligible for cisplatin-containing chemotherapy and previously received 1 or more lines of therapy (2021) ^d^
Enhertu^®^ (fam-trastuzumab deruxtecan-nxki)	HER2	IgG1	enzyme cleavable	DXd/camptothecin	TOP1 inhibitor	8	Unresectable or metastatic HER2+ breast cancer after 2 or more anti-HER2 regimens (2019) ^d^; locally advanced or metastatic HER2+ gastric or gastroesophageal junction adenocarcinoma after a trastuzumab-based regimen (2021)
Trodelvy^®^ (sacituzumab govitecan-hziy)	TROP2	IgG1	acid cleavable	SN-38/camptothecin	TOP1 inhibitor	7.6	Locally advanced or metastatic TNBC after at least two prior therapies (2020); locally advanced or metastatic urothelial cancer after a Pt-containing chemotherapy and a PD-1 or PD-L1 inhibitor (2021) ^d^
Blenrep^®^ (belantamab mafodotin-blmf)	BCMA	IgG1	non-cleavable	MMAF/auristatin	microtubule inhibitor	4	R/R multiple myeloma after at least 4 prior therapies including an anti-CD38 mAb, a proteasome inhibitor, and an immunomodulatory agent (2020) ^d^
Zynlonta^®^ (loncastuximab tesirine-lpyl)	CD19	IgG1	enzyme cleavable	SG3199/PBD dimer	DNA cleavage	2.3	R/R large B-cell lymphoma after 2 or more lines of systemic therapy, including DLBCL not otherwise specified, DLBCL arising from low grade lymphoma, and high-grade B-cell lymphoma (2021) ^d^
Tivdak^®^ (tisotumab vedotin-tftv)	Tissue Factor	IgG1	enzyme cleavable	MMAE/auristatin	microtubule inhibitor	4	Recurrent or metastatic cervical cancer with disease progression on or after chemotherapy (2021) ^d^

ADC, antibody-drug conjugate; AML, acute myeloid leukemia; B-ALL, B-cell acute lymphoblastic leukemia; BCMA, B-cell maturation antigen; cHL, classical Hodgkin lymphoma; DAR, drug-to-antibody ratio; DLBCL, diffuse large B-cell lymphoma; mAb, monoclonal antibody; MF, mycosis fungoides; MMAE, monomethyl auristatin E; MMAF, monomethyl auristatin F; pcALCL, primary cutaneous anaplastic large cell lymphoma; Pt, platinum; PTCL, peripheral T-cell lymphoma; PBD, pyrrolobenzodiazepine; R/R, relapsed and/or refractory; sALCL, systemic anaplastic large cell lymphoma; TOP1, topoisomerase I; TROP2, tumor-associated calcium signal transducer 2. ^a^ As a single agent or in combination with daunorubicin and cytarabine. Mylotarg^®^ was withdrawn from the market in 2010 and reapproved in 2017 for newly diagnosed R/R CD33-positive AML. ^b^ In combination with cyclophosphamide, doxorubicin, and prednisone for newly diagnosed sALCL or CD30+ PTCL and in combination with doxorubicin, vinblastine, and dacarbazine for newly diagnosed cHL. ^c^ In combination with bendamustine and rituximab. ^d^ Indication approved under accelerated approval.

## Data Availability

No new data were created or analyzed in this study. Data sharing is not applicable to this article.

## Appendix A

To date, seven of the FDA approved ADCs have also obtained EMA approval. These include, Adcetris^®^ (25 October 2012), Kadcyla^®^ (15 November 2013), Besponsa^®^ (28 June 2017), Mylotarg^®^ (19 April 2018), Polivy^®^ (16 January 2020), Blenrep^®^ (25 August 2020), and Enhertu^®^ (18 January 2021). Trodelvy^®^ is currently under accelerated EMA review.

## Appendix B

HR+ breast cancers are those that have cells with receptors for the hormone’s progesterone and estrogen.
